# Bacterial Virus Ontology; Coordinating across Databases

**DOI:** 10.3390/v9060126

**Published:** 2017-05-23

**Authors:** Chantal Hulo, Patrick Masson, Ariane Toussaint, David Osumi-Sutherland, Edouard de Castro, Andrea H. Auchincloss, Sylvain Poux, Lydie Bougueleret, Ioannis Xenarios, Philippe Le Mercier

**Affiliations:** 1 Swiss-Prot group, SIB Swiss Institute of Bioinformatics, CMU, University of Geneva Medical School, 1211 Geneva, Switzerland; Chantal.Hulo@sib.swiss (C.H.); Patrick.Masson@sib.swiss (P.M.); edouard.decastro@sib.swiss (E.d.C.); Andrea.Auchincloss@sib.swiss (A.H.A.); Sylvain.Poux@sib.swiss (S.P.); Lydie.Bougueleret@sib.swiss (L.B.); ioannis.xenarios@sib.swiss (I.X.); 2 University Libre de Bruxelles, Génétique et Physiologie Bactérienne (LGPB), 12 rue des Professeurs Jeener et Brachet, 6041 Charleroi, Belgium; ariane.toussaint@ulb.ac.be; 3 European Molecular Biology Laboratory, European Bioinformatics Institute (EMBL-EBI), Wellcome Trust Genome Campus, Hinxton CB10 1SD, UK; davidos@ebi.ac.uk

**Keywords:** phage, ontology, life-cycle

## Abstract

Bacterial viruses, also called bacteriophages, display a great genetic diversity and utilize unique processes for infecting and reproducing within a host cell. All these processes were investigated and indexed in the ViralZone knowledge base. To facilitate standardizing data, a simple ontology of viral life-cycle terms was developed to provide a common vocabulary for annotating data sets. New terminology was developed to address unique viral replication cycle processes, and existing terminology was modified and adapted. Classically, the viral life-cycle is described by schematic pictures. Using this ontology, it can be represented by a combination of successive events: entry, latency, transcription/replication, host–virus interactions and virus release. Each of these parts is broken down into discrete steps. For example enterobacteria phage lambda entry is broken down in: viral attachment to host adhesion receptor, viral attachment to host entry receptor, viral genome ejection and viral genome circularization. To demonstrate the utility of a standard ontology for virus biology, this work was completed by annotating virus data in the ViralZone, UniProtKB and Gene Ontology databases.

## 1. Introduction

Bacterial viruses, are the most abundant biological entity on earth. Since their discovery and the advent of molecular biology, much has been learned about their infectious cycle. Many essential discoveries in biology have been the result of bacterial virus study: not least, the identification of DNA as the molecule carrying genetic data in enterobacteria phage T2 in 1952 [1]. Bacterial viruses have proven to be potent molecular tools because they grow quickly ex vivo, their genetic material is small and manageable, and they are mostly harmless to humans. These factors contributed to put bacterial viruses at the forefront of molecular biology and promise a brilliant future for phage biotechnologies [2]. Their unique functions have provided priceless tools for biotechnology like enterobacteria phage lambda cloning, enterobacteria phage M13 sequencing [3], and recombineering [4]. An important current challenge is to monitor antibiotic resistant bacterial strains, and we know that phage therapy could be very effective in monitoring these infections. This kind of therapy is still limited in its application but has great promises [5,6].

Prokaryotic viruses comprise infectious agents for bacteria or archaea. In this manuscript we have focused on bacterial viruses because archaeal virology is complex and needs more exploration before describing in detail the molecular functions of the viruses targeting these hosts [7]. Viruses infecting bacteria are commonly called phage or bacteriophage. We prefer “bacterial viruses” denomination since people can be confused and believe that phages and viruses are different entities.

Bacterial virus biology has undergone a renaissance in recent years [8]. No longer just tools of molecular biology, these viruses are now recognized to play critical roles in bacterial pathogenesis [9], biogeochemical cycles [10], and bacterial population dynamics [11]. Moreover, new techniques in sequencing and analyses have propelled bacterial virus biology into the era of big data. These data have raised new challenges in bacterial virus genomics, proteomics, transcriptomics, and glycomics. The huge diversity of viral proteomes, their extreme number in environmental samples, and their capacity to recombine are major issues. Bacterial virus taxonomy has become more and more difficult to define and it is now clear that classical dichotomous classification does not fit bacterial viruses genomic data [12]. There is no question that bioinformatics can help to meet the challenges proposed by-omics. To do so, the knowledge available for bacterial viruses has to be available in a format compliant for computer analysis.

This work aims to bring together sequences with common knowledge in bacterial viruses biology. The UniProtKB/SwissProt virus annotation team examined the annotation and classification of all major means used by bacterial viruses to achieve their parasitic lifecycle. An extensive study of viral textbooks and literature was performed to identify the essential and conserved steps of the viral life-cycle. Despite their large diversity, bacterial viruses replication cycles can be described by a moderate number of different steps. A virus life-cycle can therefore be described by a succession of defined events. To further characterize this, we have created a controlled vocabulary comprised of 68 terms that together cover the major molecular events of a bacterial virus replication cycle.

The terms describing bacterial viruses biology were used to annotate virus entries in ViralZone [13], UniProt Knowledgebase (UniProtKB) [14] and Gene Ontology (GO) [15]. The annotation consists of associating viral sequences with controlled vocabulary, as evidenced by experimental knowledge. This requires human experts with deep knowledge of the underlying virology and a clear understanding of how to express and encode that knowledge in a consistent manner. Curators also perform an editorial function, acting to highlight (and where possible resolve) conflicting reports, one of the major added values of manual annotation. The processes identified have been developed in the form of controlled vocabulary and ontologies stored in the ViralZone, UniProtKB and GO resources.

ViralZone is a database that links virus sequences with protein knowledge using human-readable text and controlled vocabularies [13]. This web resource was created in 2009 and has been continually developed since that time by the viral curation team of the SwissProt group. The web site is designed to help people gain access to an abstraction of knowledge on every aspect of virology through two different kinds of entries; virus fact sheets and virus molecular biology pages. The latter describe viral processes such as viral entry by genome ejection and viral genome replication in detail, with graphical illustrations that provide a global view of each process and a listing of all known viruses that conform to the particular schema. ViralZone pages also provide access to sequence records, notably to the UniProtKB.

UniProtKB is a comprehensive resource for protein sequence and annotation data [14]. All known proteins are annotated in entries, either manually (Swiss-Prot) or automatically (TrEMBL). The annotation of protein function and features is assured by many means, including controlled vocabularies and ontologies. The ontologies consist of hierarchized controlled vocabulary in computer-friendly format. They provide a frame for global annotation, and facilitate the analysis of biological data. In the era of metagenomics and large-scale studies, ontologies are an extremely potent tool to link knowledge with gene products and help identify common patterns. UniProtKB keywords constitute an ontology with a hierarchical structure designed to summarize the content of an entry and facilitate the search of proteins of interest. They are classified into 10 categories: Biological process, Cellular component, Coding sequence diversity, Developmental stage, Disease, Domain, Ligand, Molecular function, Post-translational modification and Technical term.

A more complex and widely used vocabulary is the Gene Ontology (GO) in which relations between terms have a number of explicit meanings which can be used to make further inferences, such as eukaryotic transcription factors that may be located in the nucleus [15,16]. GO annotations are routinely used for the functional analysis (typically enrichment analysis) of many data types such as differential expression data. GO provides almost 40,000 terms grouped into three categories: the molecular functions a gene product performs, the biological processes it is involved in, and the cellular components it is located in. Thus far comprehensive bacterial virus biology has not been thoroughly described in this ontology. GO annotations are created manually by expert curators, as well as by automatic propagation systems. The manual curation of GO terms is a central part of the workflow at UniProtKB, and UniProt is an active member of the GO consortium. Many UniProtKB keywords are also mapped to equivalent GO terms, and the occurrence of a keyword (KW) annotation allows the annotation of the equivalent GO term (http://www.ebi.ac.uk/GOA/Keyword2GO).

## 2. Materials and Methods

This work describes the creation of a vocabulary of bacterial virus molecular biology in ViralZone, UniProtKB, and Gene Ontology. Inter-relations between vocabulary and ontologies and the way viral sequences are curated using this system have been described in a previous publication [17].

### 2.1. Creation of the Bacterial Virus Vocabulary and ViralZone Pages

As a start, all the specific steps used by bacterial viruses during their life-cycle were identified. To do so, an exhaustive study was performed of the Bacteriophage textbook [18], published reviews, and existing ontologies in GO [15] and ACLAME (A CLAssification of Mobile genetic Elements) [19] was performed.

All the processes identified were structured into six classes: virion, virus entry, latency, transcription/replication, virus release, and host-virus interactions. This led to the creation of 51 ViralZone pages describing most of the identified vocabulary (Table 1). The ViralZone pages were annotated to describe the viral processes and illustrated with a picture, and the viruses involved were listed and linked to literature references. This work is the base used to build and refine the ontologies in Gene Ontology and UniProtKB.

### 2.2. Mapping of Viral Life-Cycle Processes to GO

The GO team at the European Bioinformatics Institute (EBI) collaborated with the UniProtKB/SwissProt team to update and complete the GO database with the virus life-cycle molecular processes. The mapping effort led to the update of 24 GO terms and the development of 30 new GO terms (Table 1). Forty one of those are directly related to ViralZone vocabulary and reciprocally linked in the ViralZone and GO pages [17]. The ViralZone vocabulary does not exactly match GO ontology because the first provides general scientific knowledge, while the second defines concepts/classes used to describe gene function, and the relationships between these concepts. For example, the page “Viral penetration via permeabilization of host membrane” (VZ-985) in ViralZone describes the general process used by eukaryotic and bacterial viruses. In GO, this led to the creation of two terms because the eukaryotic and bacterialmembranes involved are not the same. The term created for prokaryotes is “viral entry via permeabilization of inner membrane” (GO:0099008), and the term for eukaryotes is “permeabilization of host organelle membrane involved in viral entry into host cell” (GO:0039665). Other terms like “Tailed bacterial virus” (VZ-4076) are concepts that cannot be strictly associated with a gene function and therefore do not lead to the creation of a corresponding GO term.

### 2.3. Creation of New UniProtKB Keywords

**Figure 1:**
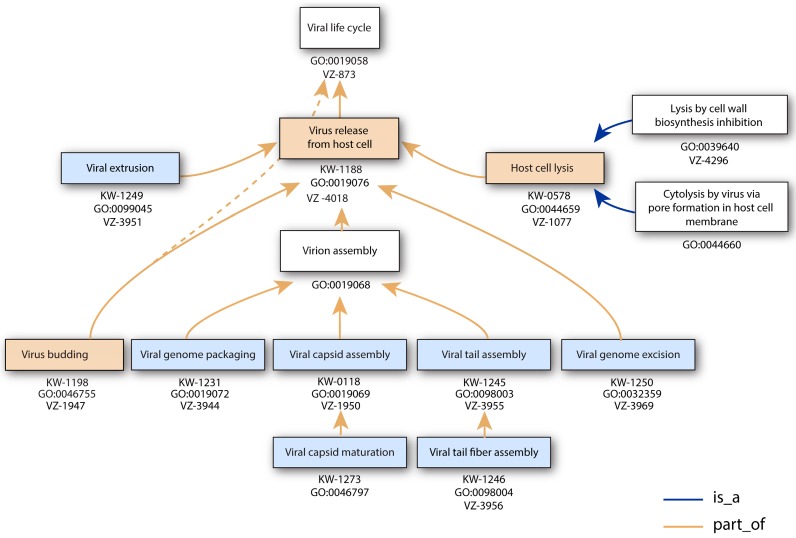
The ontology of viral release parent-child relationships. This tree consists of terms used to annotate the steps of viral release. ViralZone pages (VZ), UniProtKB keyword (KW) or GO terms accession numbers (GO:) are indicated. The hierarchy is shared by GO and KW except for budding for which the GO hierarchy is indicated with dotted lines. Boxes are colored blue for new UniProtKB KW, pink for old KW and white when the term is not related to a KW. The dotted line represents an inconsistency that will be corrected in future releases between GO and UniProt KW hierarchy: GO “virus budding” is not yet child to the “virus release from host cell” term.

Keywords (KW) summarize the content of a UniProtKB entry and facilitate the search for proteins of interest. Using ViralZone vocabulary we created 42 keywords and updated 17 KW (Table 1) for a total of 59. The keywords were developed when several different viruses use a common process that could be linked to an individual protein function. For example, the term “viral capsid maturation” was coined to annotate viral proteins whose function is to trigger capsid maturation, not to annotate the viral protein matured at that stage. UniProtKB KW and GO terms are organized in a hierarchy, an example of which is pictured in Figure 1 for virus entry.

## 3. Results

This work describes the multiple facets of bacterial virus protein functions: virion components, virus entry, host-virus interactions, viral replication and virus release.

Virus entry starts with virion attachment to the host cell, leading to the injection of the viral nucleic acid into the cytoplasm. The second step is the transcription of early viral genes, leading eventually to the replication of the viral genome. For some viruses, the onset of this first transcription step allows for a dual outcome: latency or progression to viral replication. In the first case, the viral genome is silenced after the transcription of only a few genes, putting on hold the transcription/replication step. In the second case, or when the hold is released, the viral genome proceeds to the completion of this second step without going back to latency. Other viruses always directly proceed to completion of the second step. The last step is virus release, which comprises the assembly of new particles and their release. This coincides with late transcription in most viral genomes. Often the virus will overproduce genomic and structural materials to assemble as many virions as possible. This can lead to irreversible damage to the host cell. The release of new virions is usually achieved by host cell lysis. The viral replication, assembly, and lysis are part of the virus lytic cycle. In contrast, when an integrated viral genome is passively replicated and transmitted during host mitosis, the process is called the lysogenic cycle.

In the following paragraph, viral processes discussed in the text are put between quotation marks when they correspond to a vocabulary or ontology term. The corresponding ViralZone pages can be retrieved by typing the start of the term in the ViralZone search box (http://viralzone.expasy.org/) and choosing the right name.

### 3.1. Bacterial Virions

**Figure 2:**
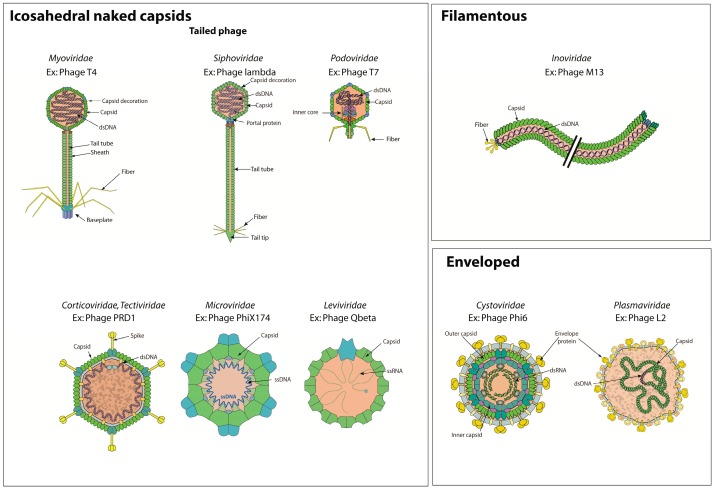
Structure of bacterial virus particles. The picture displays the different virion structures classified under three categories: icosahedral naked capsid, filamentous or enveloped particle. A representative viron structure is represented for each of the nine bacteria virus families.

Bacterial virus particles present some unique features for which we have developed a controlled vocabulary in order to annotate structural proteins. There are three kinds of bacterial virions; icosahedral naked capsid, filamentous or enveloped virion (Figure 2).

Capsids are structures protecting the viral genome, and are composed of “capsid proteins”. “Capsid decoration proteins” are located on the outermost surface of the icosahedral capsid and are involved in stabilizing the head structure. *Corticoviridae* or *Tectiviridae* capsids display an inner layer constituted by a proteinaceous lipid membrane, which envelopes the virus genome. The proteins localized in this membrane are called “capsid inner membrane proteins”. The capsid of *Cystoviridae* viruses is surrounded by a lipid membrane envelope.

The *Caudovirales* are also called “tailed bacterial viruses” because they possess an important structure (the tail) attached to a vertex of their icosahedral capsid, the function of which is to promote adsorbtion and attachment to the host cell envelope. The tail often bears a cell wall perforating device and performs genome delivery. Three families are distinguished by the morphology of their tail: *Myoviridae* (long contractile tail) [20], *Podoviridae* (short non-contractile tail) [21], and *Siphoviridae* (long flexible non-contractile tail) [22]. “Viral tail proteins” comprise all the components of the tail. “Viral tail tube proteins” are the major structural component of the tail and assemble in a tube of programmed length. In contractile bacterial viruses (*Myoviridae*), “viral tail sheath proteins” cover the tube and are responsible for tail contraction upon binding to the host receptor. This contraction induces viral DNA ejection into the host cytoplasm (see entry section below). A variable number of fibers can be attached to the tail. These “viral tail fiber proteins” are responsible for the specific, albeit reversible adsorption to the host cell. “Viral baseplate proteins” constitute the most distal part of the tail of *Myoviridae* and *Siphoviridae*. The baseplate initiates tail assembly [23], relays the contraction signal to the sheath [24] (in *Myoviridae*), and plays a role in genome ejection.

### 3.2. Virus Entry

**Figure 3:**
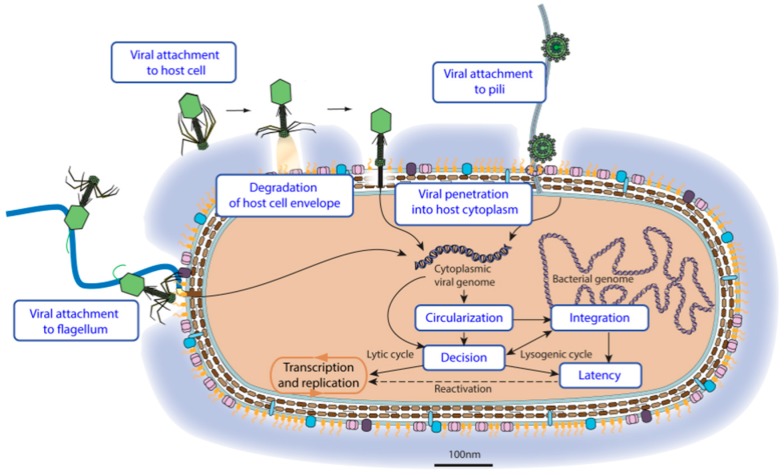
Entry pathways of bacterial viruses. This picture represents the principal ViralZone controlled vocabularies for virus entry. The representation of viral entry is chronological. The virus genome which is encapsuled in a virion on the top and left of the figure will follow alternative pathways until initiating transcription/replication processes or latency.

“Virus entry” refers to all the steps happening between the circulating virion binding to a target cell up to the delivery of viral genetic material to the site of replication or latency (Figure 3). The viral genome begins on the top of the picture and will follow alternative pathways until entering latency or the start of a lytic cycle. The nature of the virus particle plays a decisive role in the routes of entry: enveloped viruses do not face the same challenges as non-enveloped viruses. In turn, the composition of the host membranes and cell wall are determinant to the entry: crossing the cell envelope of *Mollicutes* bacteria is quite different to crossing that of Gram-positive bacteria, the envelope of which is covered by a thick glycan wall.

The first step of a virus entry is the “viral attachment to host cell”, consisting of virion interaction with the cell envelope. The binding can be reversible and is called adhesion or adsorption. “Viral attachment to host adhesion receptor” represents the initial interaction with a host receptor that positions the virus close to its target but without inducing virus entry. Adhesion can happen through various molecules present at the surface of the host cell. “Viral attachment to host cell pilus” refers to the specific adsorption to pili, which are retractile filaments up to 20 μm long that protrude from Gram-negative bacteria [25]. Some DNA bacterial viruses use host flagella to attach to the cell, a process called “Viral attachment to flagellum” [26]. The flagellum is a lash-like appendage that protrudes from the cell poles of certain bacteria.

**Figure 4:**
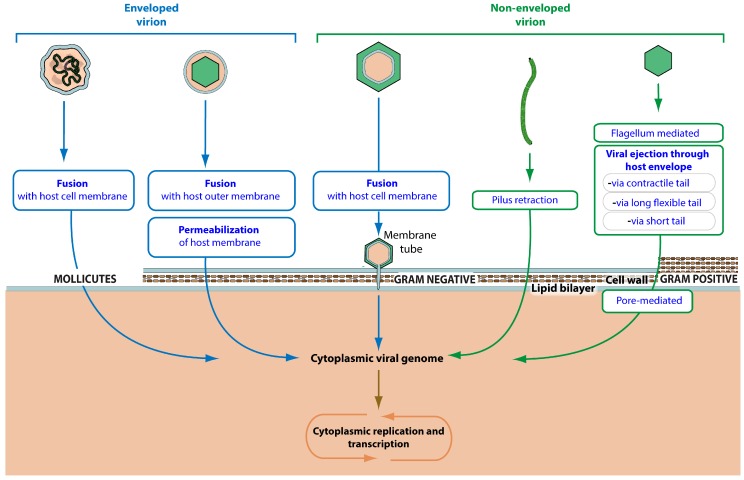
Virus crossing of the bacterial envelope. Schematic representation of different routes of envelope crossing used by bacterial viruses. The different envelopes of *Mollicutes*, Gram-positive and Gram-negative bacteria are indicated with their associated routes of entry.

Once attached to its target cell, the virus can reach an entry receptor. Binding this molecule triggers an irreversible step that leads to viral entry. “Attachment to host entry receptor” can occur at various places on the cell envelope and initiates “viral penetration into host cytoplasm” (Figure 4).

There are at least five ways for a virus to cross the bacterial envelope. Tailed bacterial viruses have developed mechanisms to trigger “viral ejection through host envelope”. These viruses are classified in families related to their ejection system: *Myoviridae* “via contractile tail”, *Siphoviridae* “Via long flexible tail” and *Podoviridae* “via short tail”. They can infect all bacteria, whatever their cell envelope.

Other virus penetration mechanisms exploit different routes of penetration depending on the nature of the host cell. Gram-negative bacteria are surrounded by two membranes separated by a peptidoglycan layer. *Tectiviridae* viruses insert a membrane tube through the host outer membrane and peptidoglycan layer to reach the cell membrane and trigger “fusion with host cell membrane”, releasing viral genomic material in the host cytoplasm [27]. An alternate route used by *Cystoviridae* viruses involves “fusion with host outer membrane”, releasing the viral capsid in the periplasmic space where it triggers the “permeabilization of host membrane” to reach the cytoplasm. Filamentous virus penetration depends on pili. The virus binds the tip of the pilus and upon “pilus retraction” the virion is brought to the inner membrane where the capsid disassembles to release the viral genomic DNA into the cytoplasm [28]. *Mollicutes* that have a simple envelope with no peptidoglycan layer are typically entered by “fusion with host cell membrane” like many eukaryotic viruses.

### 3.3. Latency

Before entering the lytic cycle or the lysogenic/latency cycle, the cytoplasmic viral genome undergoes a few more processes. A DNA virus genome can go through “viral genome circularization” [29,30], and/or “viral genome integration” into the host chromosome [31]. These events most often coincide with a crucial step called “latency-replication decision”, which depends on a molecular switch such that the virus will either enter “latency/lysogeny” or proceed to replication-assembly and lysis of the host. Latency results from the expression of regulatory and enzymatic proteins that lead to the establishment the viral genome as a silent provirus, which is replicated passively as part of the host genome. If the provirus is never reactivated, its sequence could eventually evolve as a provirus fossil. However, proviruses are also programmed for “viral reactivation from latency”. Under certain circumstances the latent genome is reactivated and initiates the transcription and replication lytic cycle. Most integrated proviruses undergo “viral genome excision” before viral replication [32].

### 3.4. Host-Virus Interactions

**Figure 5:**
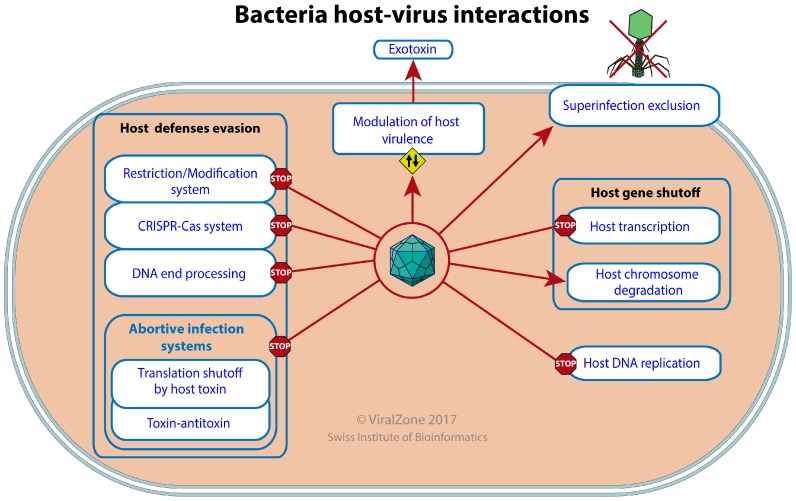
Bacterial host-virus interactions. This picture represents ViralZone controlled vocabularies for bacterial host-virus interactions. A red arrow indicates a process induced by the virus, a red line ended by a “stop” point out a process inhibited by viruses, and the up and down arrow in a yellow diamond-shape signals a process modulated up or down by viruses.

Each bacterium is the potential target of dozens of viruses, and this may be an understatement [33]. These cells have evolved efficient and complex antiviral defenses [34]. Viruses in turn have evolved elaborate mechanisms to escape, neutralize or even exploit these defenses, veritable escape artists that survive in a hostile environment [35]. We have made an extensive study of publications in order to identify the most common modes of interplay between bacterial hosts and viruses (Figure 5).

The innate cellular defenses of bacteria can induce the degradation of the infecting viral genome at the very start of the viral cycle. The restriction-modification (RM) defense [36] consists of a modification enzyme that methylates a specific DNA sequence in a genome and a restriction endonuclease that cleaves DNA lacking this methylation. Any viral genome lacking the proper methlylation will be cleaved and inactivated upon entry. Bacterial viruses have evolved different strategies for “restriction-modification system evasion”. Some viruses encode their own methyltransferase in order to protect their genome from a wide range of host restriction enzymes [35]. Enterobacteria phage T7 encodes the OCR protein that blocks the active site of several restriction enzymes by mimicking the phosphate backbone of B-form DNA [37]. Other bacterial viruses use unusual bases in their genome to avoid restriction. Bacillus phage SPO1, SP82, and 2C replace thymidine with 5-hydroxymethyluracil while in bacillus phages PBS1 and PBS2 thymine is completely changed to uracil [38].

Another bacterial defense system is DNA end degradation [39], and any bacterial virus that exposes free DNA ends upon entering the host must find a means for “DNA end degradation evasion”. Bacterial viruses have elaborated different strategies to circumvent this degradation. For example, enterobacteria phage T4 gene product 2 (gp2) is able to bind viral DNA ends to prevent their recognition by the RecBCD complex and subsequent breakdown [40]. The Gam protein of enterobacteria phage lambda also inhibits the interaction between RecBCD and viral genome ends [41].

Abortive Infection Systems (Abi) are the last host innate defense. Abi encompasses many antiviral defenses leading to host cell death, preventing further dissemination of the infecting agent [42]. Many Abi systems are mediated by cellular toxins, the activity of which can be triggered upon viral infection, thereby affecting both the virus and the host cell in an altruistic defense. The vast majority of toxins found so far interfere with translation, mostly via mRNA or tRNA cleavage. Bacterial viruses have also evolved various mechanisms to prevent this type of host defense by “evasion of bacteria-mediated translation shutoff”. A subset of antiviral toxins is part of a toxin-antitoxin system in which the toxin is normally kept inactive by the antitoxin. Bacterial viruses have evolved genes for “evasion of toxin-antitoxin system” by making up for the altered function or by mimicking the antitoxin molecule in order to protect themselves against the negative effects of toxin activation [43].

The bacterial adaptive immune defense is mediated by the CRISPR-Cas system. It relies on the ability to integrate short fragments of invading foreign DNA sequences in the form of spacers between the repetitive sequences of the CRISPR. Transcription of these sequences produces antisense RNA (crRNA) which bind and induce the cleavage of unwanted invading DNA [44]. Bacterial viruses have developed strategies for “CRISPR-Cas system evasion” [45]. For example, gene 35 from the pseudomonas phage JBD30 encodes a protein able to suppress the CRISPR system, most probably after the crRNA biogenesis. The vibrio cholerae phage ICP1 encodes its own CRISPR-Cas system that targets and silences critical antiviral genes of the bacterial host [46].

A simple way to avoid innate or acquired cellular defenses is to silence the host genetic material, a process called “host gene expression shutoff”. This shutoff not only protects the virus against most host defenses but it also ensures all the translation machinery is available to express viral proteins. We have created the UniProtKB keyword “bacterial host gene expression shutoff by virus” to discriminate between eukaryotic and bacterial processes. Silencing can be induced either by transcription inhibition or host chromosome degradation. “Bacterial host transcription shutoff by virus” is used by many viruses, most of which involve host RNA polymerase inactivation. For example the gp2 protein of T7 inhibits the correct interaction between the host RNA polymerase and the sigma transcription initiation factor [47]. “Degradation of host chromosome by virus” involves the destruction of the bacterial genetic material achieving two goals: the silencing of any antiviral response and the recycling of deoxynucleotides for viral genome replication [48,49]. Other mechanisms redirect bacterial metabolic pathways to the bacterial virus reproduction cycle. Through “Inhibition of host DNA replication”, viruses prevent host replication and division, thereby improving available dNTPs and metabolic activity for their own replication [50,51].

Most host-viral interactions are parasitic, because of the selfish nature of viral entities, but a virus cannot exist without its host; therefore beneficial interactions have also evolved that promote both virus and host survival. Many viruses that can enter latency/lysogeny protect their host cell from being infected by other similar viruses, through a process called “superinfection exclusion” [52]. This exclusion can be induced by silencing the incoming viral genome as performed by the immunity repressors of many temperate bacterial viruses including enterobacteria phage lambda [53]. Alternatively the entry of superinfecting viruses can be inhibited at the level of receptor binding [54], cell wall degradation or DNA ejection/translocation [55]. Viruses can do more than protect their host against their own kind. Being mobile genetic elements they can induce a mutualistic symbiosis by “modulation of host virulence by virus”. The latent virus can bring about a wide range of functions beneficial to its host and itself. “Viral exotoxins” (e.g., botulism toxin, diphtheria toxin, cholera toxin, and Shiga toxin, which can be found in various viruses) are secreted polypeptides that are beneficial for parasitic bacteria [56]. Bacterial viruses can also carry antigenicity modulator, intracellular survival factors, adhesion or invasion factors, or photosynthetic genes [57,58].

### 3.5. Viral Replication

Viral genome replication depends on the nature of the viral nucleic acid and comprises a wide range of specific mechanisms. Many viruses with circular double strand DNA genomes use the canonical cellular replication mechanism “dsDNA bidirectional replication” also called theta replication (circle-to-circle). This replication can be performed by viral or host DNA polymerase. “dsDNA rolling circle replication” also called sigma replication, produces long concatemers of linear genomes and requires viral enzymes. These concatemers are further processed into linear genomes for encapsidation [59,60]. Some viruses use both kinds of replication, for example enterobacteria phage lambda early replication occurs via the theta mechanisms, and later switches to rolling circle to produce the concatemers required for packaging [61]. Protein-primed replication is unique to viruses with linear dsDNA genomes and implies single-strand DNA displacement. During this “DNA strand displacement replication” only one strand is replicated at a time and the intermediate ssDNA is protected by a viral ssDNA-binding protein [62]. “Replicative transposition” is a unique mode of replication and the hallmark of transposable viruses [63]. In this process, the viral genomic DNA is first integrated into the host chromosome, then viral proteins transpose the genome from one DNA site to another, creating new copies at each transposition event [64]. The viral genomes replicated this way are later pushed into an assembled head and cleaved by viral endonucleases after the head is full. *Leviviridae* are positive stranded RNA viruses, the genomes of which and mRNA are the same molecule. These viruses undergo “viral RNA replication” through transcription by viral RNA-dependent-RNA polymerase. Replication starts similarly for double-stranded RNA *Cystoviridae*, with a supplemental step of replication within the viral capsid to synthesize the complementary RNA.

### 3.6. Virus Release from Host Cell

**Figure 6:**
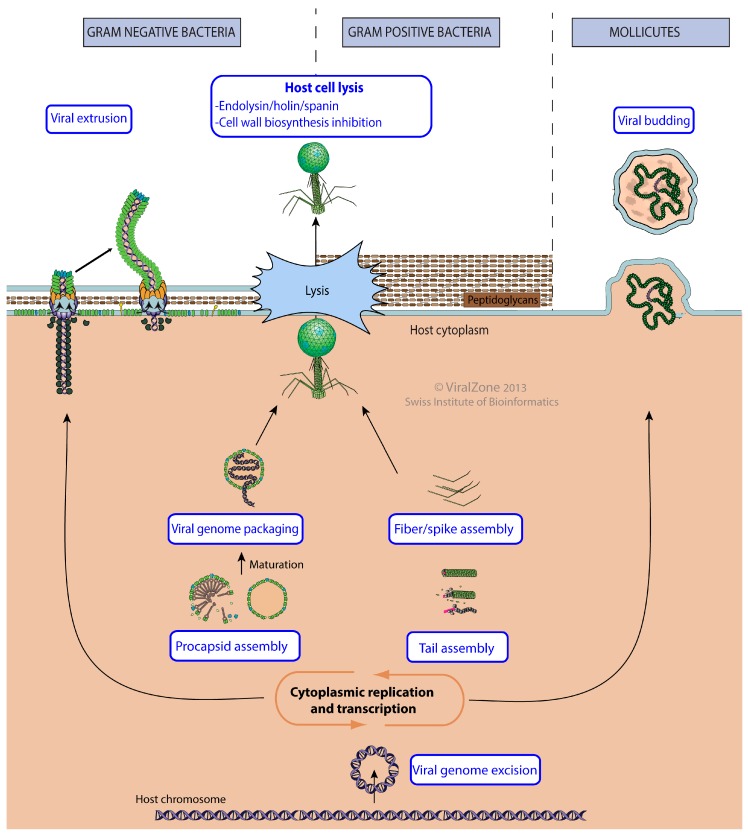
Release pathways of bacterial viruses. This picture represents the ViralZone controlled vocabulary describing the bacterial virus release pathway. The representation is chronological: The virus genome begins at the bottom of the picture after the transcription/replication processes and will follow alternative pathways until exiting the host cell at the top of picture.

The release phase is characterized by production of virion structural components and often lysis of the host to release new virions in the environment (Figure 6).

The first stage of the release consists of the virion assembly around new viral genomes. In viruses with an icosahedral symmetry, “viral procapsid assembly” creates empty particles with a portal at one vertex. Each replicated viral genome is subsequently inserted into the capsid by “viral genome packaging” through the capsid portal. For tailed bacterial viruses, this capsid will constitute the head of the virion. “Viral tail assembly” and “viral fiber assembly” occur independently. The viruses belonging to the *Corticoviridae* or *Tectiviridae* families are not tailed but have an internal membrane, which seems to be acquired during the assembly of their capsid before the packaging of the viral genome [65]. *Cystoviridae* assemble their external membrane around their capsid in the cytoplasm [66].

All virions assembled in the cytoplasm must find means to leave the host cell. This is achieved by programmed lysis of the cell by rupture of the plasma membrane. Then the osmotic pressure induces a burst of cytoplasm outside thereby releasing newly assembled virions [67]. To do so, most tailed bacterial viruses use “Holin/endolysin/spanin cell lysis” which consists of expressing lysis proteins that will accumulate at the host membrane and induce lysis by a timed mechanism independent of capsid assembly [68]. Alternatively, *Microviridae* and *Leviviridae* induce cytolysis through “cell wall biosynthesis inhibition” [69,70].

Filamentous bacterial viruses like enterobacteria phage M13 follow a different assembly procedure, called “viral extrusion” [71]. During this process, viral structural proteins are anchored in the host plasma membrane, across which the viral genome extrudes by covering itself with the capsid proteins. The budding of *Plasmaviridae* enveloped virus involves the protection of its circular DNA genome by a helical capsid, which exits the host cell by “viral budding” at the plasma membrane in ways similar to eukaryotic enveloped viruses [72].

## 4. Discussion

The virus replication cycle vocabulary and ontology have been expanded by collaboration between the UniProtKB/Swiss-Prot and GO teams. Our efforts to create bacterial virus ontology have led to three levels of implementation: global knowledge and facts in ViralZone pages; viral protein annotation in UniProtKB through keywords; viral gene and protein annotation through GO terms. Before this work, 12 KW and 26 GO terms existed, associated with few or no annotation and there were big knowledge gaps in virus life-cycle concepts. We have created 42 new SwissProt keywords, 30 new GO terms and 51 ViralZone pages to complete the existing list. Moreover we made efforts to provide annotations using this new vocabulary: at the time of writing (UniProt release 2017_04) the keywords provide a total of 2849 annotations in UniProtKB/Swiss-Prot. The future developments will be to annotate as much as possible virus sequences in order to expand the value of the bacterial virus vocabularies.

The annotation will be extended by two means that will allow annotation of existing and future big data: the InterPro to GO approach allows association of GO terms with any sequence that shares similarity with a given InterPro identifier [73]. HAMAP (High-quality Automated and Manual Annotation of Proteins) is a system for the automatic classification and annotation of protein sequences [74]. It provides annotations of the same quality and detail as UniProtKB/Swiss-Prot that are automatically assigned to virus families defined by family profiles. Those two systems will allow the dissemination of appropriate annotation across all sequences available, and provide publicly visible HAMAP virus families.

The knowledge necessary to achieve this work was not always easily accessible. Publications relevant to bacterial virus biology are spread on a wide timeframe: from 1952 to 2017. Unlike eukaryotic viruses, there are few quality textbook about bacterial viruses, and “The Bacteriophages” last edition is already 11 years old [18]. Therefore, the help of experts has been invaluable to resolve knowledge gaps, notably in complex molecular biology like replicative transposition. Eventually we managed to identify all major bacterial virus processes that allow a virus’ life-cycle to be described by a succession of controlled vocabularies. This provides a means to store and manage knowledge in biological databases. For example, the T7 virus life-cycle can be summarized by cutting this cycle into steps described by successive controlled vocabulary terms: “attachment”, “DNA ejection”, “viral transcription”, “dsDNA bidirectional replication”, “viral procapsid assembly” “viral genome packaging”, “viral tail assembly” and “host cell lysis by virus”. This succession of terms accurately describes the pathway followed by the T7 virus genome across an infected cell.

Together the ViralZone, UniProtKB and GO terms provide a global view of viral biology, and a means to associate knowledge with sequences, for a wide user community. Research groups may contribute to this viral ontology by providing suggestions for updating terms (e.g., requests for new terms) either through ViralZone (viralzone@isb-sib.ch) or Gene Ontology (http://geneontology.org/contributing-go-term). Several research institutes and public databases have initiated projects involving the annotation of viral genomes (Phagonaute [75], ACLAME and PhiGO [19], Community Assessment of Community Annotation with Ontologies CACAO [76]), and we hope that the terms and ontologies presented in this article, which are available from the ViralZone, UniProtKB and GO websites, will help them in these efforts.

## Figures and Tables

**Table 1:** Bacterial virus vocabulary. The table lists the 68 terms of the bacterial virus vocabulary as cited in the text. New terms created during this work in the three databases are indicated by a grey background. The accession numbers are indicated for GO terms GO:XXXXXXX, UniProtKB Keywords KW-XXX, and ViralZone pages VZ-XXX. The other columns indicate the number of annotations assigned to this vocabulary/ontology. The UniProtKB column displays the number of annotations made using the corresponding KW in UniProtKB bacterial virus entries (as of release 2017_04). An asterisk after a UniProtKB KW indicates a term that is also used for eukaryotic virus annotation. GO annotation lists the total number of annotation using the corresponding GO term. Terms in italics are children of the terms above them in the table.

UniProt Keywords	GO Terms	UniProt KW		ViralZone Pages	UniProt Entries
**Virion**	**GO:0019012**	**KW-0946**	*	**VZ-885**	**457**
Tailed Bacterial virus				VZ-4076	NA
Capsid protein	GO:0046728	KW-0167	*		166
*Capsid decoration protein*	GO:0098021	KW-1232			24
Viral tail protein	GO:0098015	KW-1227			138
*Viral tail sheath protein*	GO:0098027	KW-1229			11
*Viral tail tube protein*	GO:0098026	KW-1228			21
*Viral baseplate protein*	GO:0098025	KW-1226			33
Viral tail fiber protein	GO:0098024	KW-1230			42
Capsid inner membrane protein	GO:0039641	KW-1231			15
**Virus Entry into Host Cell**	**GO:0046718**	**KW-1160**	*	**VZ-3996**	**296**
Viral attachment to host cell	GO:0019062	KW-1161	*	VZ-956	72
*Viral attachment to host adhesion receptor*	GO:0098671	KW-1233	*	VZ-3943	29
*Viral attachment to host entry receptor*	GO:0098670	KW-1234	*	VZ-3942	16
*Viral attachment to host cell pilus*	GO:0039666	KW-1175		VZ-981	15
*Viral attachment to host cell flagellum*	GO:0098931	KW-1240		VZ-3949	0
Degradation of host cell envelope components during virus entry	GO:0098994	KW-1235		VZ-3938	29
*Degradation of host peptidoglycans during virus entry*	GO:0098932	KW-1236		VZ-3940	19
*Degradation of host lipopolysaccharides during virus entry*	GO:0098995	KW-1237		VZ-3939	3
*Degradation of host capsule during virus entry*	GO:0098996	KW-1238		VZ-3896	4
Viral penetration into host cytoplasm	GO:0046718	KW-1162	*	VZ-4016	161
*Fusion of viral membrane with host outer membrane*	GO:0098997	KW-1239		VZ-3941	1
*Pore-mediated penetration of viral genome into host cell*	GO:0044694	KW-1172	*	VZ-979	7
*Viral genome ejection through host cell envelope*	GO:0039678	KW-1171		VZ-986	130
*Viral contractile tail ejection system*	GO:0099000	KW-1242		VZ-3950	30
*Viral long flexible tail ejection system*	GO:0099001	KW-1243		VZ-3952	41
*Viral short tail ejection system*	GO:0099002	KW-1244		VZ-3954	32
*Viral penetration into host cell via pilus retraction*	GO:0039667	KW-1241		VZ-3953	17
*Viral penetration via permeabilization of host membrane*	GO:0099008	KW-1173	*	VZ-985	0
Viral genome circularization	GO:0099009	KW-1253		VZ-3968	8
Viral genome integration	GO:0044826	KW-1179	*	VZ-980	14
Viral receptor tropism switching	GO:0098678	KW-1264		VZ-4498	10
**Viral Latency**	**GO:0019042**	**KW-1251**	*	**VZ-3970**	**6**
Latency-replication decision	GO:0098689	KW-1252		VZ-3964	4
Viral reactivation from latency	GO:0019046	KW-1272			35
**Host–Virus Interaction**	**GO:0019048**	**KW-0945**	*	**VZ-3756**	**154**
Host defense evasion	GO:0044413				0
*Restriction-modification system evasion by virus*	GO:0099018	KW-1258		VZ-3966	16
CRISPR-Cas system evasion by virus	GO:0098672	KW-1257		VZ-3962	3
*DNA end degradation evasion by virus*	GO:0099016	KW-1256		VZ-3963	6
*Evasion of bacteria-mediated translation shutoff by virus*		KW-1259		VZ-3961	3
*Evasion of toxin-antitoxin system*				VZ-4077	0
Host gene expression shutoff by virus	GO:0039657	KW-1190	*		12
Bacterial host gene expression shutoff by virus		KW-1261		VZ-4496	12
Bacterial host transcription shutoff by virus		KW-1263		VZ-4497	4
Degradation of host chromosome by virus	GO:0099015	KW-1247		VZ-3947	8
Inhibition of host DNA replication by virus	GO:0098673	KW-1248		VZ-3948	9
Modulation of host virulence by virus	GO:0098676	KW-1254	*	VZ-3965	9
*Viral exotoxin*		KW-1255		VZ-3967	9
Superinfection exclusion	GO:0098669	KW-1260		VZ-3971	3
**Viral Replication**			*	**VZ-915**	**NA**
Viral DNA replication	GO:0039693	KW-0235	*		65
*dsDNA bidirectional replication*			*	VZ-1939	0
*dsDNA rolling circle replication*			*	VZ-2676	0
*DNA strand displacement replication*			*	VZ-1940	0
*Replicative transposition*			*	VZ-4017	0
Viral RNA replication	GO:0039694	KW-0693	*		8
**Virus Release from Host Cell**	**GO:0019076**	**KW-1188**	*	**VZ-4018**	**322**
Viral genome packaging	GO:0019072	KW-1231	*	VZ-3944	15
Host cell lysis by virus	GO:0044659	KW-0578	*	VZ-1077	79
*Lysis by cell wall biosynthesis inhibition*	GO:0039640			VZ-4296	0
*Cytolysis by virus via pore formation in host cell membrane*	GO:0044660		*		223
*Holin/endolysin/spanin cell lysis by virus*				VZ-4056	0
Viral extrusion	GO:0099045	KW-1249		VZ-3951	22
Viral genome excision	GO:0032359	KW-1250		VZ-3969	20
Viral capsid assembly	GO:0019069	KW-0118	*	VZ-1950	85
*Viral capsid maturation*	GO:0046797	KW-1273			0
Viral budding	GO:0046755	KW-1198	*	VZ-1947	0
Viral tail assembly	GO:0098003	KW-1245		VZ-3955	90
*Viral tail fiber assembly*	GO:0098004	KW-1246		VZ-3956	9
**TOTAL**					**3072**
